# Formulation, Characterisation, and Biocompatibility Assessment of Rifampicin-Loaded Poly(d,l-lactide-co-glycolide) Composites for Local Treatment of Orthopaedic and Wound Infections

**DOI:** 10.3390/pharmaceutics16111467

**Published:** 2024-11-18

**Authors:** Mitali Singhal, Colin C. Seaton, Alexander Surtees, Maria G. Katsikogianni

**Affiliations:** 1 School of Pharmacy and Medical Science, Institute of Cancer Therapeutics, University of Bradford, Bradford BD7 1DP, UK; mitali3005@gmail.com; 2 School of Chemistry and Biosciences, University of Bradford, Bradford BD7 1DP, UK; c.seaton@bradford.ac.uk; 3 School of Archaeological and Forensic Sciences, University of Bradford, Bradford BD7 1DP, UK; a.surtees@bradford.ac.uk

**Keywords:** composites, biodegradable, polymer, antimicrobial, cytocompatibility, rifampicin, PLGA

## Abstract

**Background/Objectives:** The escalating challenge of antimicrobial resistance (AMR) necessitates the development of targeted antibiotic delivery platforms, minimising systemic administration. Polymer-based drug delivery emerges as a promising solution, ensuring sustained release and prolonged efficacy of bioactive compounds, ensuring long-term efficacy. **Methods:** This study focuses on encapsulating rifampicin (RIF), a key antibiotic for orthopaedic and wound-related infections, within Poly(d,l-lactide-co-glycolide) (PLGA), a biodegradable polymer, through solvent casting, to formulate a PLGA-RIF composite membrane. Comprehensive characterisation, employing Fourier-transformed infrared spectroscopy (FT-IR), scanning electron microscopy (SEM), thermal analysis and X-ray Diffraction (XRD), confirmed the integrity of both the starting and produced materials. UV-Vis spectroscopy revealed a controlled drug release profile over 21 days in various media, with the chosen media influencing the drug release, notably the tryptic soya broth (TSB) caused the highest release. The quantitative assessment of the antimicrobial efficacy of the developed PLGA-RIF composite was conducted by measuring the size of the inhibition zones against both Gram-negative and Gram-positive bacteria. **Results:** The results confirmed the composite’s potential as a robust antibacterial biomaterial, demonstrating a rapid and effective antibacterial response. Cytocompatibility tests incorporated human fibroblast and osteoblast-like cell lines and demonstrated that the RIF:PLGA (1:8) formulation maintained eukaryotic cell viability, indicating the composite’s potential for targeted medical applications in combating bacterial infections with minimal systemic impact. **Conclusions:** This study presents the significance of investigating drug release within appropriate and relevant physiological media. A key novelty of this work therefore lies in the exploration of drug release dynamics across different media, allowing for a comprehensive understanding of how varying physiological conditions may influence drug release and its effect on biological responses.

## 1. Introduction

Currently, we are living in an ageing society and associated with this is an increased demand for prosthetic joint replacements. It has been estimated that, by 2030, there will be over 3.8 million people who will have received arthroplasties [1,2]. In the case of arthritic patients (osteoarthritis or rheumatoid arthritis), prosthetic joints are increasingly used to improve joint function and alleviate pain. Whilst most prosthetic joints are placed safely, the incidence of prosthetic joint infections (PJIs) ranges from 0.5 to 9%. Infection is the most serious complication following joint replacement, with attributable mortality rates of up to 7% in patients exceeding 80 years old [3]. Such infections are frequently problematic to diagnose and extremely challenging to treat, and often, antibiotic therapy failure occurs with recourse to surgical intervention, up to and including amputation [4,5,6].

In parallel, epidemiological studies of fracture incidence in the UK document that more than 2 million people/year suffer bone fractures that are managed using a range of medical devices [7], which in turn attract microorganisms and represent niches for medical devices and wound-associated infections [8]. External fixators are associated with ‘pin-track’ infections that have an overall incidence of about 30% [9], with the cost of individual treatment being around GBP 4000–10,000 [8]. Medical costs and the economic burden associated with PJIs are thought to be of the order of USD 8.3 billion in the USA alone [10]. Current treatment regimens are two-stage processes involving surgical removal of the joint, combined with administration of antimicrobials, followed by arthroplasty [11]. The increasing resistance of bacteria to antibiotics has stimulated interest in employing strategies for the preparation of biomaterials that resist bacterial adhesion or have bactericidal properties [12,13,14,15,16]. At present, there are no commercially available antimicrobial materials used to fabricate small joint replacements or pins.

Transitioning to the use of antibiotics topically rather than systemically offers a targeted drug delivery approach that holds the potential to enhance the therapeutic efficacy of antibiotics while simultaneously reducing the risk of potential side effects associated with widespread systemic drug distribution [17,18]. This is particularly crucial considering the growing concern surrounding the development of drug-resistant bacteria due to continuous and uncontrolled exposure to antibiotics, which can lead to the development of drug-resistant bacteria [17,19,20,21]. In this context, rifampicin (RIF), a powerful antibiotic, plays a pivotal role. RIF exhibits a broad spectrum of activity against various bacteria, encompassing both Gram-positive and Gram-negative strains [22]. Its versatility makes it a valuable weapon in combating a wide range of bacterial infections. The mechanism of action for RIF involves inhibiting bacterial RNA polymerase, a critical enzyme responsible for the synthesis of RNA and protein production in bacteria. This inhibition prevents the bacteria from multiplying and leads to their death [22,23,24]. RIF is a key component of the standard anti-tuberculosis (TB) drug, as it plays a crucial role in TB treatment due to its potency and effectiveness against Mycobacterium TB, and it is recommended by the World Health Organization (WHO) and other health authorities [25,26,27,28].

The use of biodegradable polymers for drug delivery seems like a promising approach towards the development of delivery systems for various medical applications such as sutures and fixation plates [29]. Poly(lactic-*co*-glycolic acid) (PLGA) is a biocompatible and biodegradable material [30,31]. The utilisation of PLGA conjugates represents a strategic advancement in drug delivery systems, offering distinctive advantages over alternative polymers. The intrinsic biodegradability of PLGA establishes it as the “gold standard”, ensuring sustained and controlled drug release with safety [32,33]. The capacity to finely tune the degradation rate of PLGA enables precise modulation of release kinetics, providing a tailored and patient-specific therapeutic approach. Furthermore, the versatility of PLGA extends to its ability to encapsulate a diverse array of drugs, facilitating a broad spectrum of pharmaceutical applications. The well-established clinical success of PLGA further underscores its significance as a preferred and reliable polymer in the realm of drug delivery [33]. PLGA can be formulated into nanoparticles, microparticles or films to encapsulate RIF [25,34,35].

The aim of this study was to produce composite materials that would provide a controlled and sustained release of RIF from the PLGA matrix over an extended period and to explore the effect of various media on the RIF release profile while also investigating the influence of diverse media on the RIF release kinetics. The controlled release would allow for a sustained therapeutic effect, reducing the dosing frequency and enhancing patient compliance [28,36]. RIF can be sensitive to environmental factors, such as light and moisture, which might degrade its potency over time [37]. PLGA encapsulation can protect the drug from these external influences, increasing its stability and shelf life [38]. RIF, like many antibiotics, may cause adverse effects when administered systemically. Localised delivery using PLGA can help reduce systemic exposure, thus minimising potential side effects on other organs or tissue.

PLGA nanoparticles have been engineered to target specific sites of infection, improving drug accumulation at the site of action [28]. This study aimed to develop films that can be of any thickness, rather than nanoparticles, that can be further processed for suture or fixation applications and to study the release of RIF from a fully dense composite material in order to optimise the RIF loading in PLGA and explore how the RIF release is affected by various media. The ultimate aim is to maintain a sustained and optimal concentration of RIF delivery through PLGA [38]. Therefore, this project aimed to tune biomaterial compositions by encapsulating two different concentrations of RIF in PLGA. The materials were characterised using physicochemical, antibacterial and cytocompatibility testing and the effect of the various media on the drug release so that antibacterial and cytocompatibility properties are better understood and linked to the released RIF concentration.

## 2. Materials and Methods

### 2.1. Materials

Phosphate-buffered saline (PBS) tablets (P4417) and PLGA (430471) with molecular weights of 50,000–75,000 were purchased from Sigma Aldrich (Dorset, UK). RIF was obtained from EMD Millipore Corp (Dorset, UK). All chemicals were used without any further purification. All solvents (HPLC grade) were obtained from Fisher Scientific (Loughborough, UK).

Petri dish, culture flasks and 96 well plates were purchased from Sarsted (Leicester, UK). Recombinant Trypsin–EDTA 1X, penicillin/streptomycin 100X, l-glutamine 100X and foetal bovine serum (FBS) were purchased from Lonza Biologics (Basel, Switzerland). Dulbecco’s modified Eagle’s medium (DMEM) was purchased from Sigma Aldrich (Dorset, UK). 3-(4,5-dimethylthiazol-2-yl)-2,5-diphenyltetrazolium bromide (MTT) was sourced from MERCK-Sigma (Dorset, UK). Human dermal fibroblast (HDF) cells were procured from Caltag Medsystems (Buckingham, UK), and human osteosarcoma cells (MG63) were obtained from Merck (Dorset, UK).

### 2.2. Synthesis of PLGA—RIF Composites via Solvent Casting

In a beaker, 200 mg of PLGA pellets were dissolved in 15 mL of chloroform, and the solution was stirred for 30 min until the pellets were fully dissolved. Separately, for the RIF:PLGA (1:2) weight ratio, 100 mg of RIF was weighed, and for the RIF:PLGA (1:8) weight ratio, 25 mg of RIF was used. The weighed RIF was then added to the PLGA-chloroform solution under continuous stirring for an additional 20 min. Subsequently, the mixtures were allowed to set overnight in silicone moulds, during which the chloroform evaporated, resulting in the formation of composite membranes.

### 2.3. Material Characterisation

The material characterisation was conducted using various analytical techniques. Fourier transform–infrared (FT-IR) analysis was performed in the range of 650–3500 cm^−1^ using a Perkin-Elmer Spectrum 100 ATR FT-IR Spectrometer. Scanning electron microscope (SEM) images were obtained using a FEI Quanta 400 SEM instrument under vacuum conditions. Prior to SEM analysis, the samples were sputter-coated with gold using a Sputter Coater K550X (Emitech, Quorum Technologies Ltd., London, UK). EDS analysis was conducted using the SEM system fitted with an Oxford Xplore30 EDS after the samples were carbon sputtered. Thermogravimetric analysis (TGA) was performed using a TGA Instruments Q5000IR. The temperature of the samples gradually increased from 10 °C to 600 °C at a heating rate of 5 °C min^−1^, under a nitrogen purge gas flow of 25 mL/min. Differential scanning calorimetry (DSC) measurements were performed with a [TA DSC Q2000] calorimeter. The temperature of the samples increased from 0 °C to 150 °C at a heating rate of 5 °C min^−1^, and the data were processed with the TA instruments Universal Analysis 2000 software. X-ray diffraction (XRD) was recorded at ambient temperature using a Bruker D8 diffractometer, 2θ range 10–40°, step size of 0.01°, with reflection geometry using a Cu Kα_1_ (λ = 1.54056 Å) source and Lynxeye detector. UV-Vis spectrometer (Perkin Elmer, Waltham, MA, USA) was used to assess the drug release concentration, as detailed in the section below (Section 2.4).

### 2.4. Drug Release Studies

The PLGA-RIF polymer samples (30 mg) were immersed in 30 mL of different release media, including phosphate-buffered saline (PBS) at pH 7.4, Tryptic Soy Broth (TSB), and Dulbecco’s modified Eagle’s medium (DMEM), all pre-conditioned at 37 °C. Subsequently, the samples were kept at a constant temperature of 37 °C using a shaking incubator at 100 rpm.

For the release studies, 3 mL of the supernatant was extracted from each vial at various time points, up to 21 days, to monitor the release of RIF. To ensure sink conditions, the extracted supernatant was promptly replaced with an equal volume of fresh media after each sampling. The concentration of RIF released at each time point was quantitatively determined using UV-Vis spectrophotometry. RIF showed λmax at 335 nm and 475 nm [39] (Figure S2). A calibration plot was established in PBS, TSB and DMEM using standard solutions, and the absorbance of each dilution was measured at 475 nm (Figure S3). The concentration (*Ct_corr_*) of RIF released from the PLGA-RIF composite at different time points, in triplicates, was calculated using Equation (1).

(1) $${Ct}_{corr}=Ct+\frac{v\sum_{c=0}^{t-1}Ct}{V}$$

where *Ct* is the calculated concentration of RIF at the specific time point using the calibration curve. *v* is the volume of the extracted sample (3 mL in this case). *V* is the total volume of the release solution.

This equation considers the cumulative effect of previously extracted samples on the corrected concentration at each time point. By summing the concentrations of RIF from all previous time points up to the current time point and adding it to the calculated concentration (*Ct*), the corrected concentration (*Ctcorr*) is obtained.

### 2.5. Antimicrobial Analysis

#### 2.5.1. Direct Antimicrobial Activity

The antimicrobial analysis was conducted to evaluate the effectiveness of the PLGA, RIF and PLGA-RIF (1:8) and (1:2) composites against both Gram-positive and Gram-negative bacterial strains, namely *S. aureus* NCTC 6571, *S. epidermidis* NCIMB 8853, *E. coli* NCTC 12923 and *Acinetobacter baumannii* ATCC 19606. The zone of inhibition assay was employed, using 1 mg of the PLGA-RIF composites, RIF and PLGA for comparison. Bacterial cultures were prepared overnight on Tryptic Soya Agar and then diluted in sterile water to achieve a turbidity of 5 × 10^8^ colony forming units (CFUs) mL^−1^, as per the McFarland standard [40]. These bacterial suspensions were spread on Mueller–Hinton agar and the weighed samples of PLGA-RIF composites were placed on agar plates. After incubating the plates for 24 h at 37 °C, the diameter of the inhibition zones surrounding the samples was measured to determine the antimicrobial activity.

#### 2.5.2. Non-Direct Antimicrobial Activity

In addition to the direct antimicrobial evaluations, supplementary tests were conducted to assess the antimicrobial activity of the released RIF from the composite materials by immersing the samples (1 mg) in TSB (1 mL) at 37 °C. Bacterial suspensions were spread on punched agar plates, and 50 μL of the release solution collected after 1 h, 6 h, and 24 h was placed on agar plates and incubated for 24 h at 37 °C. The resulting inhibition zones were measured as indicators of the potential antimicrobial effects stemming from the released RIF in TSB.

### 2.6. Cytocompatibility Analysis

The cytocompatibility analysis adhered to the ISO 10993-12-2021 standard [41], utilising fibroblasts [42,43,44] and osteoblast-like cells [45,46,47] as common cell models, consistent with established practices in similar studies. Human dermal fibroblasts (HDFs) and osteosarcoma (MG63) cells were chosen as the cell models and cultured in high-glucose DMEM supplemented with 10% foetal bovine serum (FBS), 2 mM l-glutamine, 100 U/mL penicillin, and 0.1 mg/mL streptomycin. The cell culture was maintained in a humidified environment at 37 °C with 5% CO_2_. The cells were regularly passaged when the cell confluence reached 70–80%.

#### 2.6.1. Non-Direct Cytocompatibility Testing

For non-direct cytocompatibility analysis, membrane extracts were prepared. Sterilisation was performed by subjecting the samples to 20 min of UV irradiation. To prepare the membrane extracts, 100 mg of the RIF loaded and unloaded samples were taken and immersed in 1 mL of cell culture media. Subsequently, these samples were placed at 37 °C for 24 h, as per the ISO 10993-12-2021. These extracts were then used for cytocompatibility assessments with the HDF and MG63 cells.

Briefly, the cells were seeded at a concentration of 10^5^ cells mL^−1^ of complete high-glucose DMEM. The resulting cell suspension was dispensed into 96-well cell culture plates. The plates were then incubated for 24 h to enable the cells to adhere and proliferate. Subsequently, the culture media were replaced by the loaded and unloaded membrane extracts for the following 24 h. Controls, including positive (DMSO) and negative (untreated cells), were incorporated. The MTT assay was employed to quantify cell number and viability. Briefly, 1 mg mL^−1^ MTT solution was added for 3 h incubation. After removing the MTT solution, isopropyl alcohol was added, the plates were shaken at 100 rpm for 10 min and the absorbance was measured at 570 nm (Flexi Station microplate reader).

#### 2.6.2. Direct Cytocompatibility Assay

The direct cytocompatibility assay assessed the cytotoxicity of antibiotic-loaded PLGA, adhering to the ISO 10993-12-2021 for in vitro cytotoxicity. Composite samples of 1 mg were prepared and sterilised using UV. MG63 cells were seeded into a 96-well plate at a density of 10^5^ cells mL^−1^ and incubated for 24 h to form a monolayer. After 24 h, a sterile 1 mg sample was placed at the centre of the dish over the cell monolayer, ensuring continuous contact for another 24 h. Controls, including positive (DMSO) and negative (untreated cells), were incorporated. Subsequently, the composite materials were removed, the dishes washed with PBS and the MTT assay, as detailed in the non-direct cytotoxicity assay (Section 2.6.1), was performed.

### 2.7. Statistical Analysis

The statistical analysis of the data was conducted using ANOVA two-way, Holm–Sidak utilising the GraphPad software, Version 8 For each experimental assay, triplicate measurements were performed in parallel. The results are presented as the mean value ± standard deviation (SD). In this analysis, significance was determined for *p*-values less than 0.05.

## 3. Results and Discussion

### 3.1. Characterisation of Materials

The integration of RIF into the polymer resulted in a distinct orange hue, indicating a homogeneous distribution of the antibiotic within the PLGA matrix.

#### 3.1.1. FT-IR

**Figure 1:**
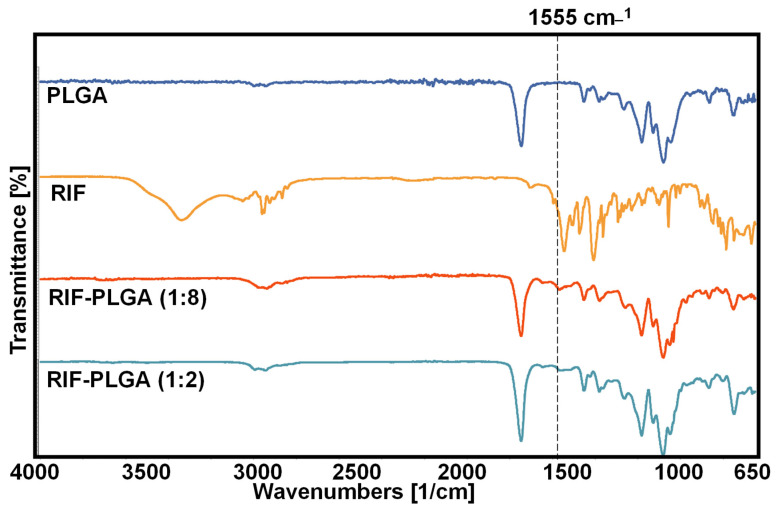
FTIR spectra for PLGA, RIF, RIF:PLGA (1:8) and RIF:PLGA (1:2).

FT-IR spectra of PLGA composites are shown in Figure 1. The spectra agree with the published ones for RIF and PLGA [48,49,50,51]. Regarding PLGA, the bands found at 2944 cm^−1^ and 2866 cm^−1^ represent asymmetrical and symmetrical CH_2_ stretching, the one band found at 1721 cm^−1^ is indicative of C=O carbonyl stretching, and the peak at 1293 cm^−1^ represents C—O and C—C stretching. The bands at 1239 cm^−1^ and 1165 cm^−1^ are attributed to asymmetrical and symmetrical stretching of O-C-O bonds, respectively.

The FT-IR spectrum of RIF exhibited prominent peaks at 3478, 2937, 1726, 1248, and 894 cm^−1^, corresponding to −OH, −CH_3_, C = O, C−O−C functionalities. Remarkably, the FT-IR spectrum of the PLGA-RIF composites shows indications of RIF loading in PLGA, with identifiable bands aligning with peaks from the RIF spectra, notably one highlighted at around 1555 cm^−1^ (Figure 1). The FTIR structure of PLGA-RIF is more closely related to PLGA. This similarity in spectra may be indicative of a strong interaction between RIF and PLGA, substantiating the effective encapsulation of RIF within the PLGA matrix. FTIR for all samples, including the aged ones in various solutions, is presented in Figure S1.

#### 3.1.2. SEM

**Figure 2:**
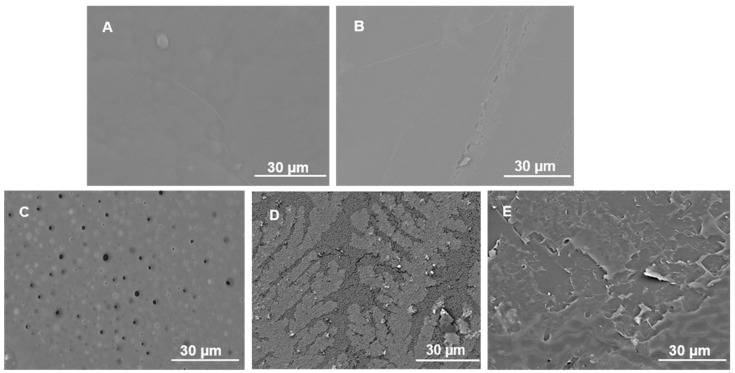
SEM images before immersion, including (**A**) PLGA and (**B**) PLGA-RIF. Following 21 days of immersion, SEM images of PLGA-RIF are presented for (**C**) TSB-immersed composites, (**D**) DMEM-immersed composites and (**E**) PBS-immersed composites.

The SEM image of the PLGA surfaces (Figure 2A) aligns with previously reported observations and presents a smooth surface [52]. The RIF-loaded film exhibited a notably flat appearance, and no discernible indications of RIF were visibly present on the surface of the composite (Figure 2B). The flat morphology observed post-loading could imply a homogenous distribution of RIF within the PLGA film. The PLGA composite morphology was influenced by the immersion in various media, namely PBS, TSB and DMEM. The SEM images of the PLGA composites revealed notable differences after 21 days of immersion, indicating signs of degradation of PLGA [53]. Notably, SEM images of RIF-PLGA in TSB (Figure 2C) showed the presence of pore-like structures, which correlated with a higher release of RIF (data presented in Section 3.2) compared with DMEM (Figure 2D) and PBS (Figure 2E).

#### 3.1.3. EDS

The EDS analysis (Table 1) of the PLGA sample revealed a composition dominated by carbon and oxygen, consistent with the expected elements in PLGA. The introduction of RIF into PLGA resulted in an increase in carbon content, indicative of successful drug incorporation. The presence of nitrogen in the PLGA-RIF sample confirms the addition of RIF. Following immersion, the composition shifted slightly, with an increase in oxygen content. This change might be attributed to interactions with the immersion media affecting the surface. Moreover, after immersion of the PLGA-RIF samples, the nitrogen becomes non-detectable, showing drug release (Table 1 and Figure S6). DMEM immersion introduced additional elements (sodium and aluminium), suggesting interactions with the media components. The overall stability of the PLGA-RIF composite in DMEM, 21 days post immersion, is indicated by minimal compositional changes but drug release.

**Figure 3:**
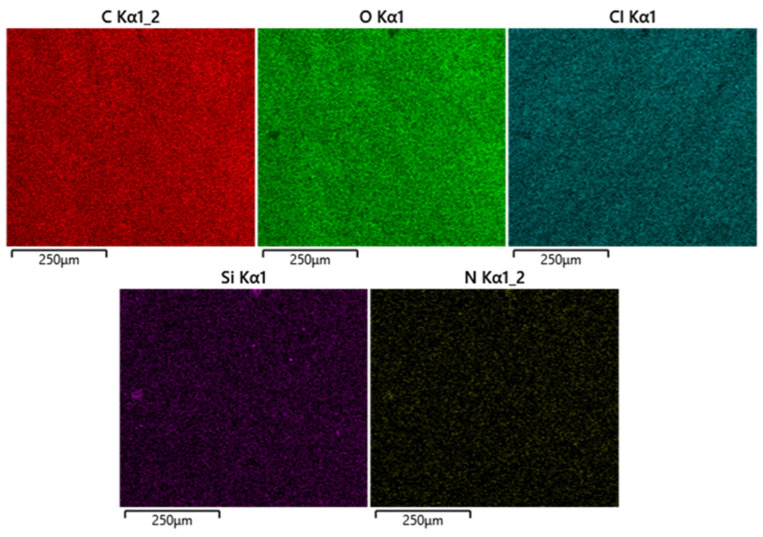
Elemental composition mapping of PLGA-RIF composite prior to immersion. Figure S6 in the supplementary presents the EDS mapping of the other samples; PLGA and PLGA immersed and PLFA-RIF immersed samples.

Figure 3 illustrates the uniform dispersion of RIF within the PLGA matrix prior to immersion, as indicated by the uniform distribution of nitrogen.

#### 3.1.4. TGA

**Figure 4:**
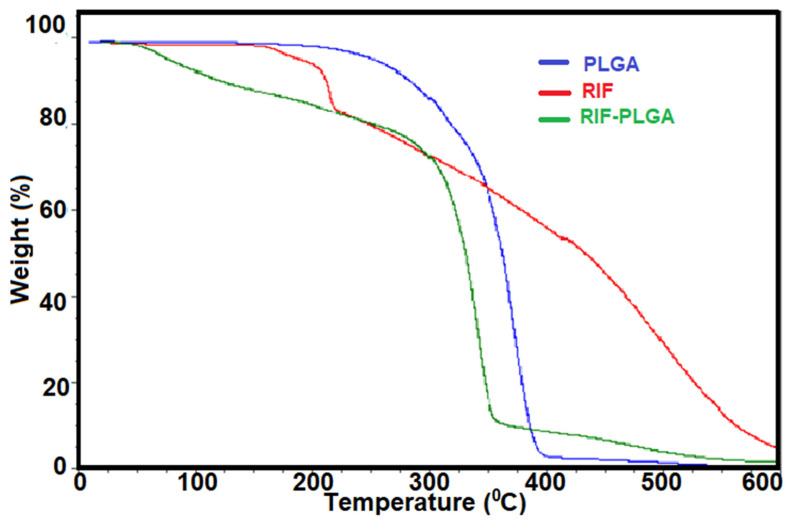
TGA for PLGA, RIF and RIF:PLGA (1:8) composite.

TGA was employed to investigate the thermal stability of all tested materials. The findings (Figure 4) revealed the influence of the PLGA matrix on the thermal stability [49,52,54] of the composite. The thermal analysis indicated that free RIF underwent initial decomposition at 200 °C [26], while the PLGA-RIF composite displayed delayed decomposition onset at 350 °C. This delay in decomposition onset strongly suggests a protective effect conferred by the PLGA matrix on the incorporated RIF. The thermal stability imparted by the PLGA matrix underscores its role as a robust protective barrier, shielding the encapsulated RIF from premature degradation. This protective effect is attributed to the barrier properties of PLGA, retarding the access of heat and degradation agents to the encapsulated RIF. These results offer valuable insights for applications involving RIF and biodegradable composites, informing material selection and processability.

#### 3.1.5. DSC

**Figure 5:**
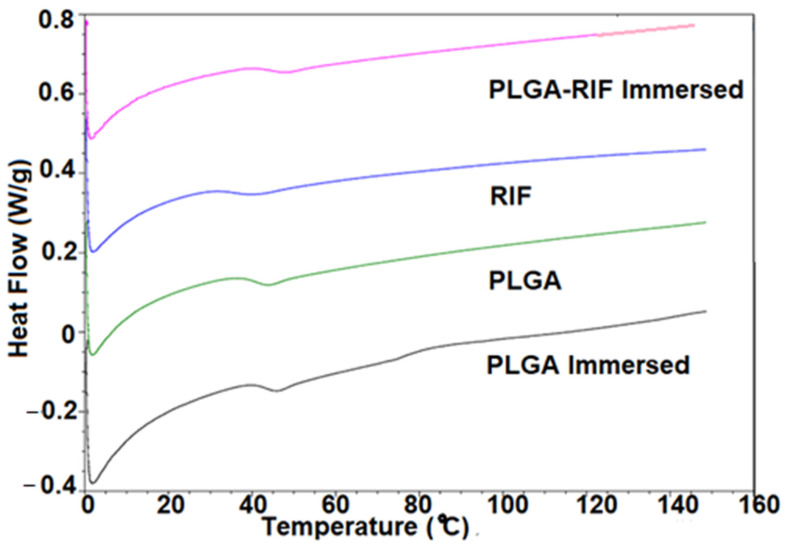
Glass transition temperatures of PLGA (with and without Immersion), RIF and PLGA-RIF (after immersion in DMEM) composite.

The presence of RIF in PLGA-RIF-immersed materials tends to shift the glass transition temperature (T_g_) to higher values compared with their PLGA counterparts (Figure 5). This shift suggests potential interactions between RIF and PLGA, affecting the overall thermal behaviour. Immersion appears to influence the thermal properties, as seen in the differences among PLGA, RIF and immersed PLGA-RIF. The changes in T_g_ parameters indicate alterations in the polymer matrix, potentially due to water absorption and other environmental factors during immersion. Comparing the T_g_ values (Table 2), it is evident that PLGA-RIF has a lower T_g_ than PLGA-RIF immersed. This difference suggests that immersion may induce changes in the polymer structure, affecting its thermal transitions.

The graph illustrates the glass transition temperatures (T_g_) of PLGA and PLGA-RIF under different conditions, including immersion. “PLGA-RIF immersed” represents T_g_ values after immersion, “PLGA-RIF” indicates T_g_ values without immersion, “PLGA” shows T_g_ values of PLGA and “PLGA immersed” represents T_g_ values of PLGA after immersion. The data demonstrate variations in T_g_ under different conditions, providing insights into the thermal behaviour of the materials.

#### 3.1.6. Powder X-Ray Diffraction (PXRD)

**Figure 6:**
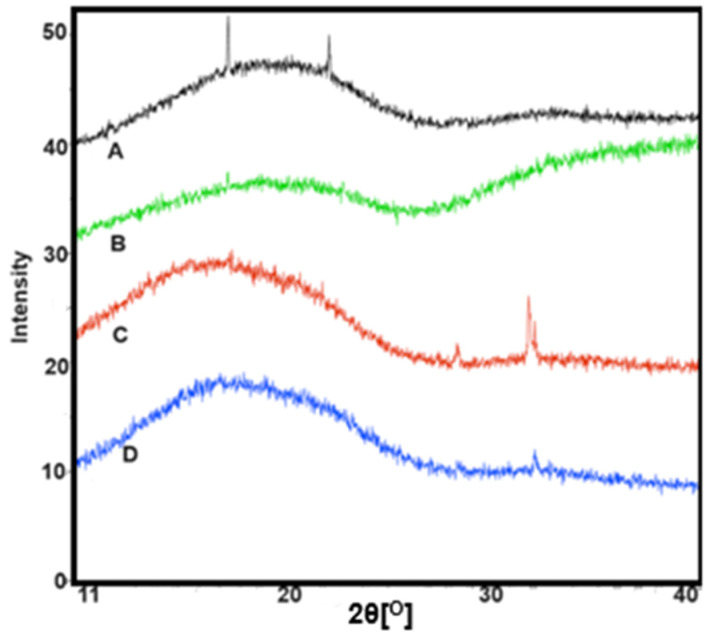
Comparison of XRD patterns for (A) pure PLGA and (B) PLGA-RIF before immersion; (C) pure PLGA and (D) PLGA-RIF after immersion in DMEM.

XRD analysis of the four samples (PLGA, PLGA-RIF, each before and after immersion) shows varying levels of crystallinity in the samples (Figure 6). For pure PLGA, both samples display a level of crystallinity with sharp diffraction peaks atop the amorphous curve. The change in the location of the peak positions indicates a shift in the crystal structure. The addition of RIF generates a predominately amorphous phase, but following immersion, a crystallinity similar to that of the pure phase is obtained. The level of crystallinity is highest for the pure PLGA after immersion, which reflects the largest shift in T_g_ values.

### 3.2. Drug Release Studies

**Figure 7:**
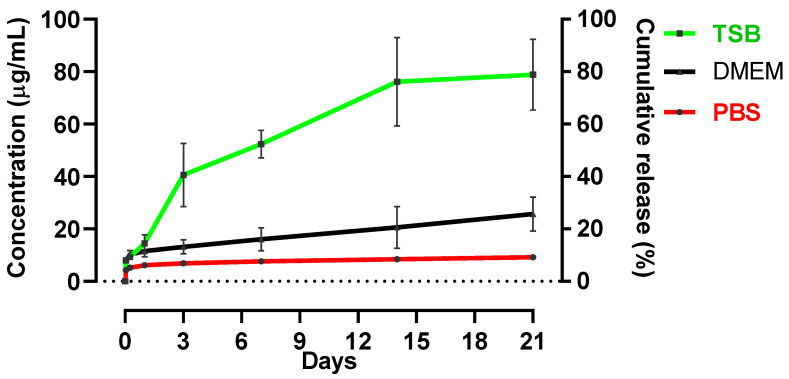
The kinetics of RIF delivery from RIF-PLGA (1:8) composite in different media (PBS, TSB and DMEM) at 37 °C over a period of 1 h to 21 days. Each data point represents the average ± standard deviation (SD) obtained from three independent experiments.

The release kinetics of RIF from the RIF-PLGA composite with a 1:8 weight ratio was investigated over a 21-day period (Figure 7). Three distinct media environments were employed: PBS at pH 7.4, TSB and DMEM. These conditions aimed to replicate physiological settings at a constant temperature of 37 °C, akin to body temperature.

The release profiles exhibited significant variations across the different media. In PBS, the composite demonstrated a gradual and sustained release similar to what has been shown in the literature for various PLGA-RIF formulations [24,38]. RIF concentrations reached 8.42 ± 0.52% after 14 days. This pattern bears similarity to RIF release by poly (D,L-lactide) microspheres in PBS at pH 7.4, where equilibrium was achieved after 15 days [24,55].

In DMEM, a release profile similar to PBS was observed. The initial release at 1 h was 8.14 ± 0.48%, and subsequent time points exhibited incremental increases in released RIF concentrations. The composite maintained a steady release, reaching 25.73 ± 6.5% in 21 days. Notably, sustained RIF release for up to 6 weeks has been reported in vivo using PLGA microparticles in a murine model [28].

This release profile agrees with the tri-phasic release profile, which is possibly the most common for PLGA [56,57]. Phase I in the classic tri-phasic release profile is usually described as a burst release and has been attributed to non-encapsulated drug particles on the surface or drug molecules close to the surface and easily accessible by hydration. Phase II is often a slow-release phase, during which the drug diffuses slowly, either through the relatively dense polymer or through the few existing pores, while polymer degradation and hydration proceed. Phase III is usually a period of faster release, often attributed to the onset of erosion. This phase is sometimes called the second burst. In our case, we did not observe Phase III in the case of PBS and DMEM, as the study concluded 21 days post immersion, and according to the literature, Phase III takes place soon after [56,57].

In contrast, the TSB medium presented a distinct trend; the composite displayed a tri-phasic release with a short phase II release pattern [56,57]. Within the first hour, the released RIF concentration was 8.1 ± 0.59%. Over time, this cumulative release escalated to a maximum of 78.86 ± 7.06% at 21 days. No similar study has been found in the literature using TSB as release media.

The data, therefore, underscore the composite’s ability to adapt its release kinetics to different media environments. While PBS induced a slow and sustained release [24], TSB prompted an accelerated release, and DMEM exhibited an intermediate pattern. These findings highlight the profound influence of the surrounding environment on drug release from the composite because of differences in degradation and diffusion [55]. It is, therefore, important to tailor drug delivery systems to specific applications and conditions. Such insights pave the way for the development of more precise and effective drug delivery strategies, catering to diverse therapeutic requirements and enhancing the efficacy of pharmaceutical interventions.

The minimum inhibitory concertation (MIC) of RIF depends on the bacterial strains against which it is tested. According to the literature, the MIC for *S. aureus* is ≤1 μg mL^−1^ [58,59,60,61]. Similarly, for *E. coli*, the MIC has been found at 4 μg mL^−1^ [62], and *A. baumannii* exhibits an MIC range of 2–4 μg mL^−1^ [60,63]. Moreover, for *S. epidermidis,* the MIC has been found at 0.25 μg mL^−1^ [59]. When considering drug delivery systems, maintaining RIF concentrations at or above these MIC values is paramount to ensuring its therapeutic effectiveness and mitigating the risk of antibiotic resistance [61]. The concentrations observed in release studies (Figure 7) align with and fall within the range of these therapeutic concentrations, making the composite system an ideal candidate for the development of biodegradable implants targeted at specific infection sites.

### 3.3. Antimicrobial Analysis

The data in Table 3 present the zones of inhibition of the direct influence of PLGA-RIF composites on the antibacterial activity against four bacterial strains: *E. coli*, *S. aureus*, *S. epidermidis* and *A. baumannii*, when the composites were placed directly on the agar plates.

The antimicrobial properties of all components were assessed by the zone of inhibition assay. The RIF was more effective against Gram-positive bacteria than Gram-negative ones (Table 3). This supports previous research [58], which states that RIF is more effective against Gram-positive bacterial strains such as *S. aureus*, as it has an MIC of ≤1 μg mL^−1^ [59,60,61] in comparison to *E. coli*, which has an MIC value of 4 μg mL^−1^ [62]. This insight enhances the understanding of the differential antimicrobial effectiveness of RIF against distinct bacterial types [58].

The results reveal a comprehensive picture of how different composite compositions affect bacterial growth. Analysing the outcomes for *E. coli*, a clear trend emerges wherein the antibacterial efficacy of the composites increases with higher RIF concentrations. Notably, RIF exhibited an inhibition zone of 30.2 ± 1 mm, the highest among all tested formulations, which was expected due to its direct contact with the bacteria. In contrast, within the composite formulations, RIF is encapsulated and released gradually, resulting in a slower onset of antimicrobial activity (Figure S4). The PLGA also exhibited a zone of inhibition measuring 7.6 ± 0.5 mm, which was notably enhanced to 15 ± 1 mm when the RIF:PLGA (1:2) composite was introduced, further reaffirming the potent antibacterial effect of RIF. However, at a lower RIF concentration, as seen in the RIF:PLGA (1:8) composite, the inhibition zone dropped to 4 ± 1 mm. The effect of PLGA against bacterial growth indicates that PLGA has some inherent antimicrobial activity [24].

Similar trends are observed for *S. aureus* and *S. epidermidis*. The RIF:PLGA (1:8) composite consistently displayed substantial antibacterial activity, outperforming the PLGA-only composite. However, the RIF:PLGA (1:2) composite demonstrated slightly enhanced efficacy against *S. aureus* compared with the RIF:PLGA (1:8) composite, while for *S. epidermidis*, the difference in antibacterial activity between the two RIF concentrations was marginal.

Interestingly, *A. baumannii* exhibited variations in response. RIF demonstrated the highest inhibition zone of 60 ± 1.2 mm, while the PLGA composite displayed an inhibition zone of 13.3 ± 2.8 mm and the RIF:PLGA (1:2) composite demonstrated a notably higher zone of inhibition at 25 ± 1 mm. However, the zone of inhibition slightly decreased to 20 ± 0 mm with the RIF:PLGA (1:8) composite.

The antibacterial efficacy of RIF-PLGA composites is, therefore, profoundly influenced by composite composition and bacterial strains. It is also interesting to observe that the decrease in RIF-PLGA ratio from 1:2 to 1:8 only slightly reduced the zones of inhibition for most bacterial strains, as observed in Table 3, and this can be justified by the MIC release in Tryptic Soya-based media, as suggested by the release profile in Figure 7. These findings contribute to the growing body of knowledge regarding composite-based antibacterial strategies, offering insights into the optimisation of composite formulations for specific bacterial targets and how testing in the appropriate media is of great importance.

The results obtained from the non-direct inhibition of bacterial growth (Table 4), as well as the release study, present a compelling correlation between the released RIF concentrations and the observed inhibition zones for different bacterial strains.

In the context of *E. coli*, both PLGA and RIF-PLGA composites demonstrated limited inhibition zones throughout the tested durations, reflecting the absence of significant antimicrobial effects. This aligns with the fact that this bacterial strain is more resistant to RIF. However, for *S. aureus*, *S. epidermidis* and *A. baumannii*, a noteworthy pattern emerges. PLGA exhibits intrinsic antibacterial activity likely due to its surface properties, biodegradation products creating an acidic microenvironment and prevention of microbial attachment [64,65,66,67,68]. The RIF-PLGA composites consistently exhibited larger inhibition zones compared with PLGA alone across all bacterial strains and time points, as expected [24].

Specifically, the correlation between RIF release and antibacterial activity is presented for each bacterial strain, as shown below.

*E. coli*: At 1 h, despite an 8.14% RIF release, RIF-PLGA only managed an inhibition zone of 2 mm. As the RIF concentration elevated to 10.2% at 6 h and 10.7% at 24 h, there was a marginal increase in the inhibition zone, reaching 3 mm, suggesting that the released RIF concentration might be close to *E. coli*’s threshold of susceptibility.

*S. aureus*: An intriguing trend was noticed. At 1 h with 8.14% RIF release, the inhibition zone was already a pronounced 29 mm, which remained consistent even as the RIF release increased to 10.7% at 24 h. This suggests that even the initial RIF release from the composite was substantially effective against *S. aureus*, indicating its high susceptibility to RIF [24,38].

*S. epidermidis*: At the 1 h mark with an 8.14% RIF release, the inhibition zone was 32 mm, which was further augmented to 34 mm by 24 h with a 10.7% RIF release. The increase in zone diameters correlates with the gradual RIF release, underlining the composite’s prolonged efficacy [24,38].

*A. baumannii*: A similar progressive pattern was seen. With an 8.14% RIF release at 1 h, an inhibition zone of 20 mm was observed, which expanded to 24 mm by the 24 h mark as the RIF release touched 10.7%. The increasing zone diameters suggest a direct correlation with the escalating RIF concentrations over time (Figure S5). The consistent increase in RIF release from the composite, along with the inherent antibacterial activity of PLGA, seems to be the reason for the observed increasing antibacterial activity for PLGA-RIF against the tested bacterial strains. While the release rate of RIF was gradual, the inhibition zones, especially for RIF-PLGA, were consistently large for bacteria like *S. aureus*, *S. epidermidis* and *A. baumannii* in comparison to *E. coli*.

### 3.4. Cytocompatibility

**Figure 8:**
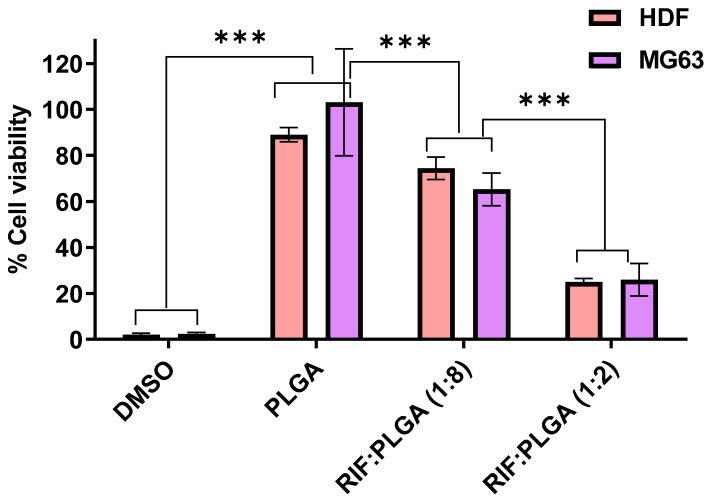
Indirect cytotoxicity profile on HDF and MG63 after treatment with 20% DMSO, PLGA, RIF:PLGA (1:8) and RIF:PLGA (1:2). The data shown represent average ± SD of 3 independent experiments normalised to the control value of cell line without treatment, which was set at 100%. (*** *p* < 0.001).

The non-direct cytotoxic effects of PLGA and PLGA-RIF composites, through their extracts, on HDF and MG63 cells have been presented in Figure 8. The control group exhibited a cell viability of 100%, indicating the baseline viability of untreated cells. The 20% DMSO positive control confirmed the cytotoxic effect of DMSO on both cell lines. Considering the PLGA, the relative cell viability remained above 90% for both cell lines, indicating that the PLGA at 100 mg mL^−1^ concentration had a minimal impact on cell viability. PLGA undergoes hydrolysis in the body, producing the original monomers, lactic acid, and glycolic acid, that can be efficiently managed by the body’s metabolic pathways, thereby minimising systemic toxicity and potentially contributing to cytocompatibility [34]. The PLGA maintaining a high cell viability signifies its biocompatibility, aligning with established standards for biomaterial safety. This is a crucial characteristic of biomaterials intended for use in medical devices and implants [38,69,70,71].

The RIF:PLGA (1:8) composite demonstrated minimal cytotoxic effects, maintaining cell viability at approximately 70% in both HDF and MG63 cells. This finding is promising, suggesting that the composite has the potential for use in applications where maintaining high cell viability is essential, such as in medical devices or implants.

On the contrary, the RIF:PLGA (1:2) composite exhibited significantly reduced cell viability, dropping to approximately 25% in both cell lines. Although this ratio may possess potent antimicrobial effects, the observed cytotoxicity raises concerns about its broader biomedical applications. It suggests that optimisation strategies are needed to enhance the safety profile of the composite, ensuring its suitability for use in various medical contexts.

This considerable decrease in cell viability indicates higher cytotoxicity at this ratio (1:2) but also the significant reduction in RIF release in the DMEM media, in comparison to TSB (Figure 7), showing that this reduction from the RIF:PLGA 1:2 to RIF-PLGA 1:8 is significant enough in DMEM to affect cell viability, but not significant enough in TSB to affect antibacterial activity, with the 1:8 still being antibacterial but not cytotoxic. These results underscore the critical role of composite composition in influencing cell viability. The RIF:PLGA (1:8) composite stands out as a more biocompatible option, holding promise for safer and more effective biomaterials in the realm of biomedical applications. This study contributes to the ongoing efforts to develop advanced materials with enhanced biocompatibility, addressing a key aspect in the design and optimisation of medical devices and drug delivery systems [36,70,72].

**Figure 9:**
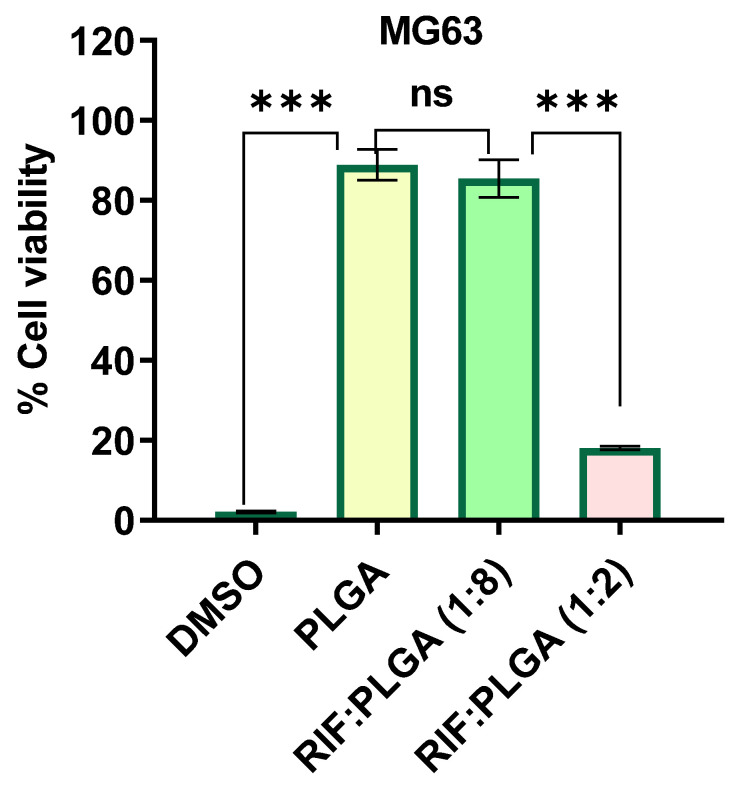
Direct cytotoxicity profile on MG63 after treatment with 20% DMSO, PLGA, RIF:PLGA (1:8) and RIF:PLGA (1:2). The data shown represent average ± SD of 3 independent experiments normalised to the control value of cell line without treatment, which was set at 100%. (*** *p* < 0.001, ns—non-significant).

The direct cytotoxicity profile on MG63 cells, as depicted in Figure 9, reveals distinct differences between the 1 mg samples of PLGA and PLGA-RIF. Notably, the cytotoxicity of RIF:PLGA (1:2) is observed to be higher compared to RIF:PLGA (1:8). This discrepancy in cell viability between the non-direct and direct cytocompatibility tests for MG63 cells suggests that the specific cell line might influence the release kinetics of the drug from the composites.

The selection of the MG63 cell line in this study adheres to the customary practice of employing osteosarcoma cell lines as a surrogate model for osteoblast-like cells [45,46,47]. However, it is acknowledged that this choice may not entirely capture the behaviour of primary human osteoblasts. Therefore, for future investigations, a comparative analysis evaluating the impacts on both osteosarcoma and normal osteocytes would offer a more holistic understanding of how these biomaterials influence distinct cell types.

This study specifically focuses on encapsulating RIF within a PLGA composite membrane, presenting a distinctive targeted drug delivery platform. This novel delivery approach offers localised and sustained release, minimising the systemic impact in contrast to conventional systemic administration. This research showcases potential applications in preventing biofilm formation associated with medical implants and treating infections in musculoskeletal injuries. The prevention of biofilm formation is a critical aspect addressed uniquely by this study, addressing a key challenge in managing infections linked to medical implants. The comprehensive characterisation techniques employed, including FT-IR, SEM, TGA, XRD, EDS and UV-Vis spectroscopy, contribute to a thorough examination of the developed PLGA-RIF composite, providing detailed insights into its structural, thermal and optical properties.

The quantitative assessment of antimicrobial efficacy involves measuring inhibition zones against both Gram-negative and Gram-positive bacteria, surpassing traditional antibacterial evaluations. Additionally, the study goes beyond conducting cytocompatibility testing on human fibroblast and osteoblast-like cell lines, expanding the evaluation to hint at the composite’s potential for targeted medical applications with reduced systemic impact. This dual approach establishes a layer of biological relevance and safety assessment, setting it apart from the nanoparticle-based approach in the published paper and highlighting potential clinical applications by considering both antimicrobial efficacy and biocompatibility.

Unlike the published papers, this research investigates the controlled drug release profile over 21 days in various media, with emphasis on the effect of various media on drug release, providing novel insights into the composite’s behaviour under diverse conditions. The significant release observed in TSB highlights the importance of evaluating drug release in relevant media rather than just in PBS, which is commonly done in many studies. This is particularly pertinent since the human body is not solely composed of PBS. Thus, our emphasis on different media adds a valuable dimension to understanding how biological observations should be linked to environmental properties affecting the material’s behaviour. Future studies would benefit from incorporating mechanical and swelling testing to further elucidate these relationships.

## 4. Conclusions

In this study, the biodegradable polymer PLGA was successfully loaded with the antimicrobial agent RIF, ensuring the efficient formulation of the composite materials. Elemental composition analysis, through EDS, not only confirmed successful drug incorporation but also demonstrated the homogeneous distribution of RIF within the composite film. The thermal stability assessed by TGA revealed a protective effect conferred by PLGA, delaying RIF decomposition and showing its role as a barrier against premature drug release. According to XRD, the addition of RIF generated a predominately amorphous phase, but following immersion, a similar crystallinity was obtained to the pure phase 21 days post immersion, showing that crystallinity is reversible after drug release. The level of crystallinity was highest for the pure PLGA after immersion, which reflects the largest shift in T_g_ values. The controlled drug release studies in various media environments showcased the adaptability of the composite’s kinetics, a pivotal feature for tailored drug delivery. The release study demonstrated that PLGA-RIF exhibited a gradual and extended-release profile in DMEM, followed by PBS, while in TSB, more of a burst release was observed due to the porosity that was introduced by the presence of TSB, as observed by SEM. The sustained rise in drug concentration over a 21-day period aligns with the study’s objective of achieving prolonged and localised drug release, particularly relevant for musculoskeletal wound infections. The observed increase in inhibition zones over time, consistent across different bacterial strains, underscores the composite’s ability to deliver an effective concentration of RIF, inhibiting bacterial growth. Pure RIF exhibited a significantly higher zone of inhibition compared with the composites, which can be attributed to its direct contact with the bacterial strains. However, the composite forms of RIF provided controlled and sustained release, which is advantageous for prolonged antimicrobial effects. The comparative analysis of two different ratios of RIF in the composite revealed better cytocompatibility for the RIF:PLGA (1:8) ratio, coupled with a prompt and sustained antimicrobial impact on various microbes. This aligns with our initial objectives and presents a potential strategy to control the rise in antimicrobial resistance by offering sustained drug delivery. These findings hold promise for the development of antimicrobial strategies utilising the RIF-PLGA composite as a controlled release system, making a significant contribution to the field of biomedical materials and infection control. While this study has provided valuable insights, ongoing research aims to further extend drug delivery and explore the cytocompatibility of composite materials using primary osteoblasts.

This comprehensive approach, combining material characterisation, drug release kinetics, antimicrobial efficacy and cytocompatibility, positions the PLGA-RIF composite as a versatile and effective candidate for targeted drug delivery, showcasing its potential clinical relevance in managing infections associated with medical implants and musculoskeletal injuries. The integration of biofilm prevention as a focal point further distinguishes this study, addressing a critical challenge in the field and reinforcing the potential of this composite in diverse therapeutic applications.

## Figures and Tables

**Table 1:** Elemental composition analysis of PLGA-based composites before and after immersion.

Element	PLGA wt%	PLGA-RIF wt%	PLGA (DMEM Immersed) wt%	PLGA-RIF (DMEM Immersed) wt%
C	56.91	59.36	57.22	56.97
N	---	1.11	---	
O	39.17	36.35	42.31	41.7
Si	0.85	0.21	---	
Cl	3.07	2.97	0.47	0.88
Na	---	---	---	0.18
Al	---	---	---	0.26
Total	100	100	100	100

**Table 2:** Glass transition temperatures (T_g_) of PLGA and PLGA-RIF under different conditions.

Material	*T_g_* Onset (°C)	*T_g_* Mid (°C)	*T_g_* Offset (°C)
PLGA-RIF immersed	48.7	52.3	52.6
RIF	42.3	45.7	46.9
PLGA	46.5	46.8	48.7
PLGA immersed	46.9	48.3	50.1

PLGA-RIF immersed: glass transition temperatures of PLGA-RIF after immersion, RIF: glass transition temperatures of RIF, PLGA: glass transition temperatures of PLGA, PLGA immersed: glass transition temperatures of PLGA after immersion.

**Table 3:** The direct antimicrobial activity of PLGA, RIF and PLGA-RIF composites against various bacterial strains measured as zones of inhibition after 24 h of incubation.

Bacteria-Materials	PLGA (mm)	RIF (mm)	RIF:PLGA (1:2) (mm)	RIF:PLGA (1:8) (mm)
*E. coli*	7.6 ± 0.5	30.2 ± 1	15 ± 1.2	4 ± 1
*S. aureus*	16.6 ± 2.8	60 ± 0.5	43 ± 1	41.3 ± 2.3
*S. epidermidis*	22.6 ± 2.5	66.1 ± 2.8	50 ± 0.9	44.6 ± 0.5
*A. baumannii*	13.3 ± 2.8	60 ± 1.2	25 ± 2.7	20 ± 0

**Table 4:** Non-direct antimicrobial activity of the PLGA and PLGA-RIF composites after 1 h, 6 h and 1 day of immersion, measured as zones of inhibition after 24 h of incubation.

Bacteria-Material	PLGA (mm)			RIF:PLGA (1:8) (mm)		
Time	1 h	6 h	1 day	1 h	6 h	1 day
*E. coli*	0 ± 0	0 ± 0	0 ± 0	2 ± 0.5	3 ± 0.5	3 ± 0.5
*S. aureus*	9 ± 1	15 ± 1.5	20 ± 2	27 ± 1.5	30 ± 1.5	30 ± 2
*S. epidermidis*	4 ± 1.1	6 ± 0.5	9 ± 1.1	32 ± 1.5	34 ± 2.5	35 ± 1
*A. baumannii*	8 ± 0.5	10 ± 1.1	13 ± 2	21 ± 1	24 ± 2	24 ± 2

## Data Availability

The data presented in this study are available on request from the corresponding author.

## Supplementary Materials

The following supporting information can be downloaded at: https://www.mdpi.com/article/10.3390/pharmaceutics16111467/s1, Figure S1. FTIR spectra for (A) PLGA:RIF (2:1), (B) PLGA:RIF (8:1), including pure PLGA, RIF. Additionally, it presents FTIR spectra of PLGA and PLGA-RIF composite immersed in different media, namely PBS, TSB and DMEM, for 21 days. PLGA (PLGA alone), RIF (RIF alone), PLGA-PBS (PLGA immersed in PBS), PLGA-TSB (PLGA immersed in TSB), PLGA-DMEM (PLGA immersed in DMEM), PLGA-RIF (composite), PLGA-RIF-PBS (PLGA-RIF immersed in PBS), PLGA-RIF-TSB (PLGA-RIF immersed in TSB), PLGA-RIF-DMEM (PLGA-RIF immersed in DMEM). Figure S2: RIF peaks by UV-Vis spectroscopy. Figure S3: A. RIF calibration curve in PBS. Figure S3: B. RIF calibration curve in TSB. Figure S3: C. RIF calibration curve in DMEM. Figure S4: The direct antimicrobial activity of PLGA, RIF and PLGA composites against bacterial strains after 24 h. Figure S5: Non-direct antimicrobial activity of the PLGA and PLGA-RIF composites after 1 h, 6 h and 1 day of immersion in TSB and 50 µm of the supernatant against bacteria. Figure S6: EDS- (A), PLGA (B), PLGA-RIF (C), PLGA Immersed in DMEM (D), PLGA-RIF Immersed in DMEM.
