# Supplementary material for: Formulation, Characterisation, and Biocompatibility Assessment of Rifampicin-Loaded Poly(d,l-lactide-co-glycolide) Composites for Local Treatment of Orthopaedic and Wound Infections
Source: Pharmaceutics. 2024 Nov 18;16(11):1467. doi: 10.3390/pharmaceutics16111467 (PMC11597898; doi:10.3390/pharmaceutics16111467)
Supplement: Supplementary file 1 [file pharmaceutics-16-01467-s001.zip › pharmaceutics-3244834-supplementary.pdf]

Formulation, characterisation, and biocompatibility assessment of Rifampicin-loaded Poly(d,l-lactide-co-glycolide) composites for local treatment of orthopaedic and wound infections

*Mitali Singhal [1], Colin C. Seaton [2], Alexander Surtees [3], Maria G. Katsikogianni[2]**

[1] School of Pharmacy and Medical Science, University of Bradford, Bradford, BD7 1DP, United Kingdom.

[2] School of Chemistry and Biosciences, University of Bradford, Bradford, BD7 1DP, United Kingdom.

[3] School of Archaeological and Forensic Sciences, University of Bradford, Bradford, BD7 1DP, United Kingdom.

* Correspondence: m.katsikogianni@bradford.ac.uk; Tel.: +44-(0)1-2742-36185

Keywords: Composites, biodegradable, polymer, antimicrobial, cytocompatibility, rifampicin, PLGA

Supplementary material

Samples of PLGA and RIF-PLGA after 21 days of immersion used for FTIR

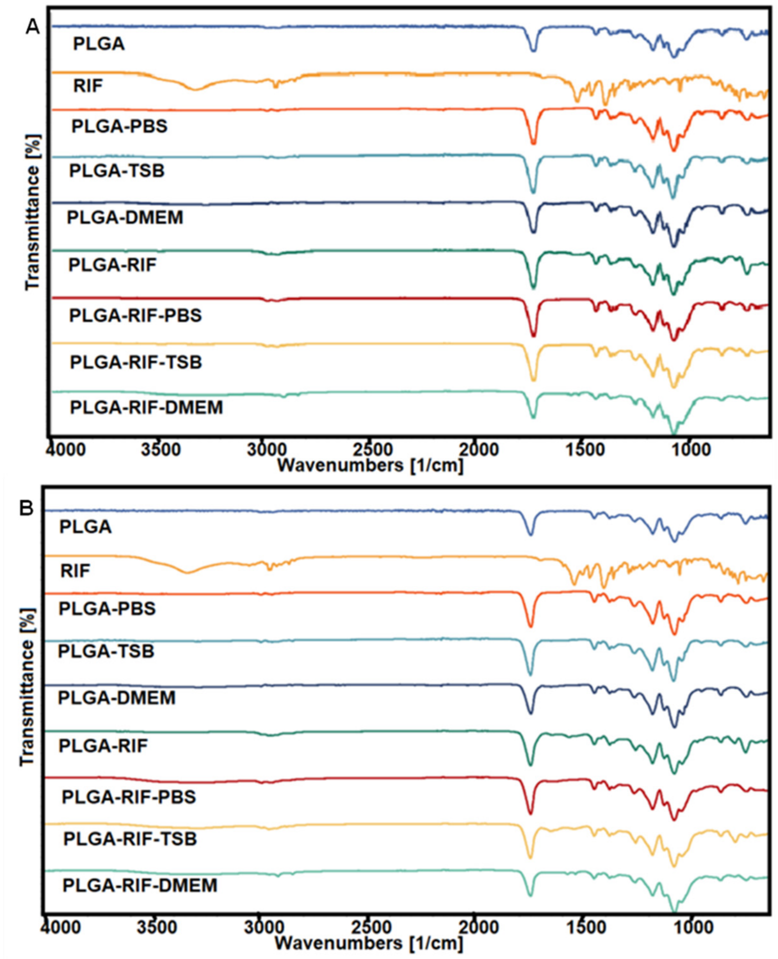

Figure S1. FTIR spectra for (A) PLGA:RIF (2:1) (B) PLGA:RIF (8:1), including pure PLGA, RIF. Additionally, it presents FTIR spectra of PLGA and PLGA-RIF composite immersed in different media, namely PBS, TSB, and DMEM, for 21 days. PLGA (PLGA alone), RIF (RIF alone), PLGA-PBS (PLGA immersed in PBS), PLGA-TSB (PLGA immersed in TSB), PLGA-DMEM (PLGA immersed in DMEM), PLGA-RIF (composite), PLGA-RIF-PBS (PLGA-RIF immersed in PBS), PLGA-RIF-TSB (PLGA-RIF immersed in TSB), PLGA-RIF-DMEM (PLGA-RIF immersed in DMEM).

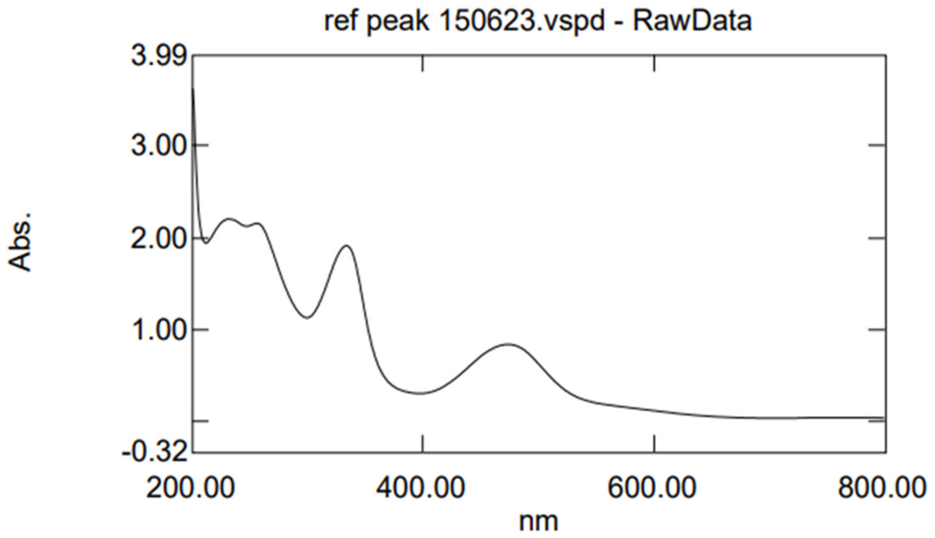

Figure S2: RIF peaks by UV-Vis spectroscopy

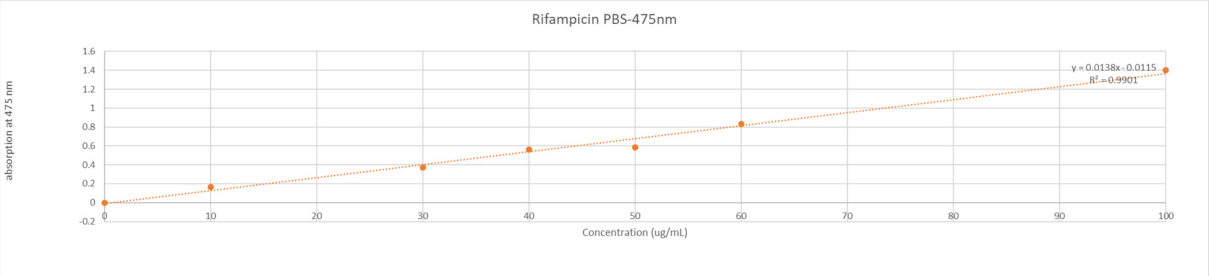

Figure S3: A. RIF calibration curve in PBS

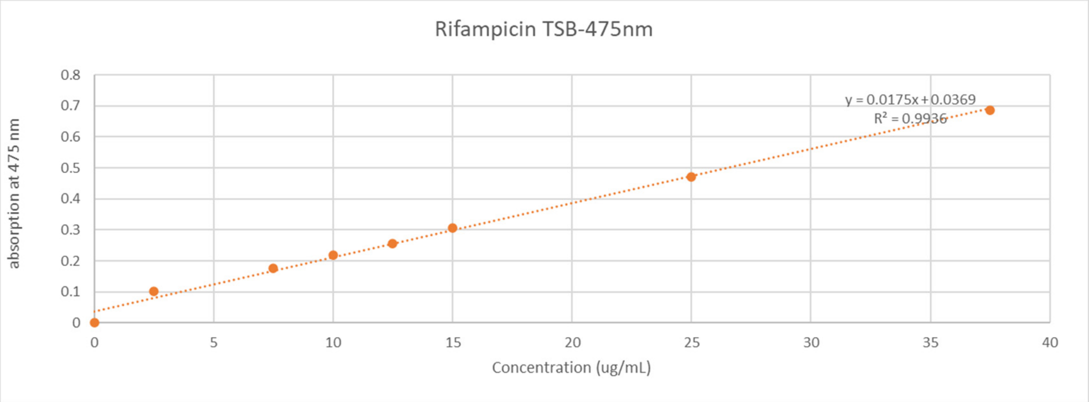

Figure S3: B. RIF calibration curve in TSB

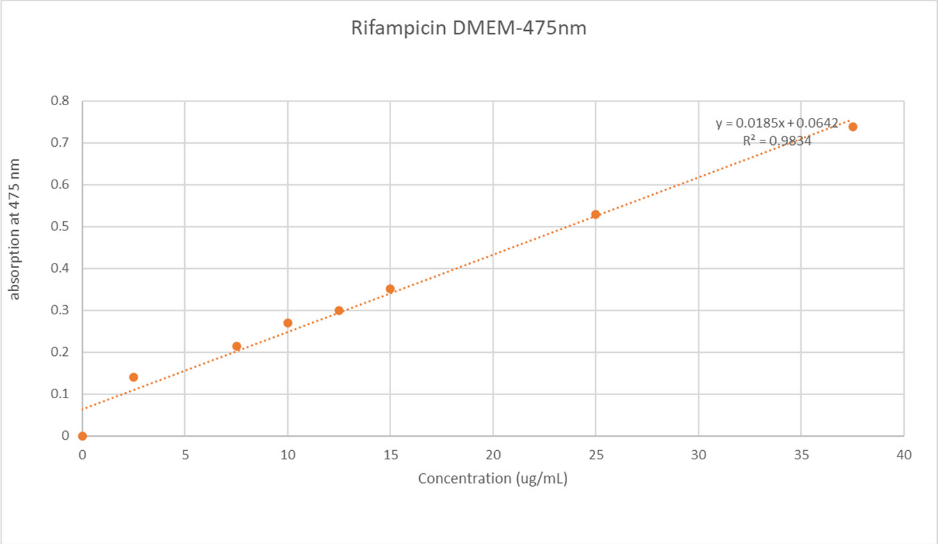

Figure S3: C. RIF calibration curve in DMEM

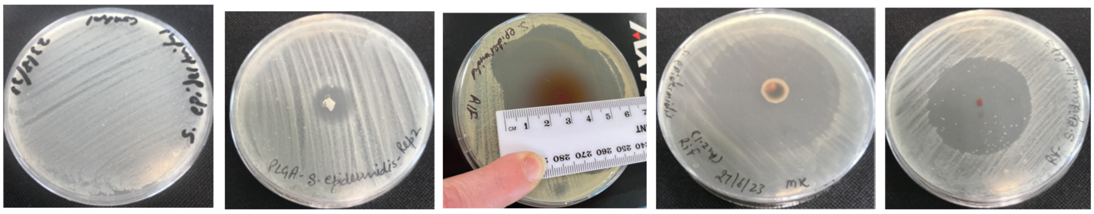

Control PLGA RIF PLGA:RIF (2:1) PLGA:RIF (8:1)

S. epidermidis

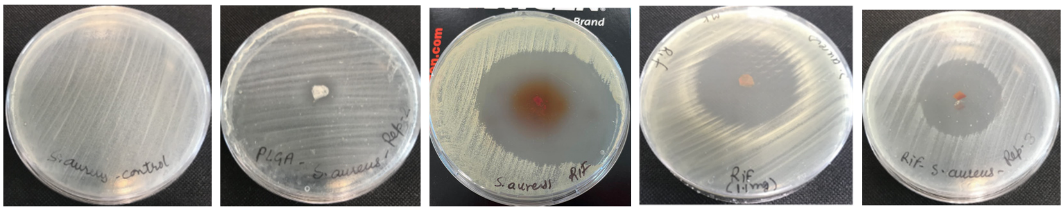

Control PLGA RIF PLGA:RIF (2:1) PLGA:RIF (8:1)

S. aureus

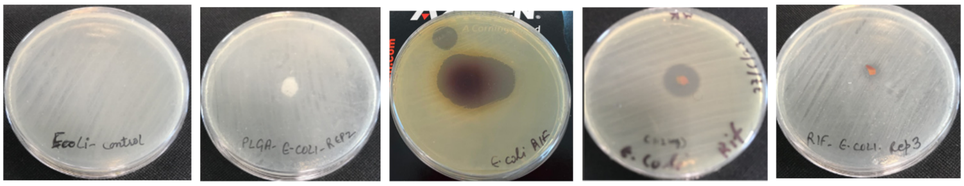

Control PLGA RIF PLGA:RIF (2:1) PLGA:RIF (8:1)

E. coli

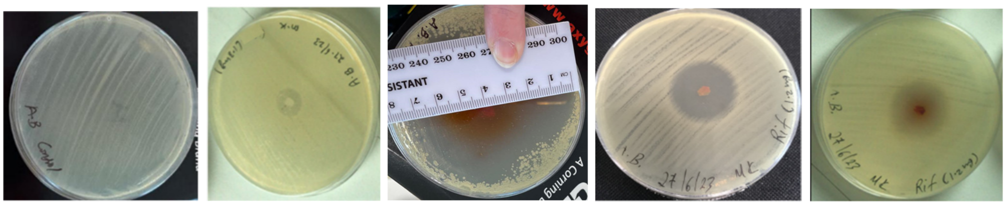

Control PLGA RIF PLGA:RIF (2:1) PLGA:RIF (8:1)

A. baumannii

Figure S4: The direct antimicrobial activity of PLGA, RIF, and PLGA composites against bacterial strains after 24 h.

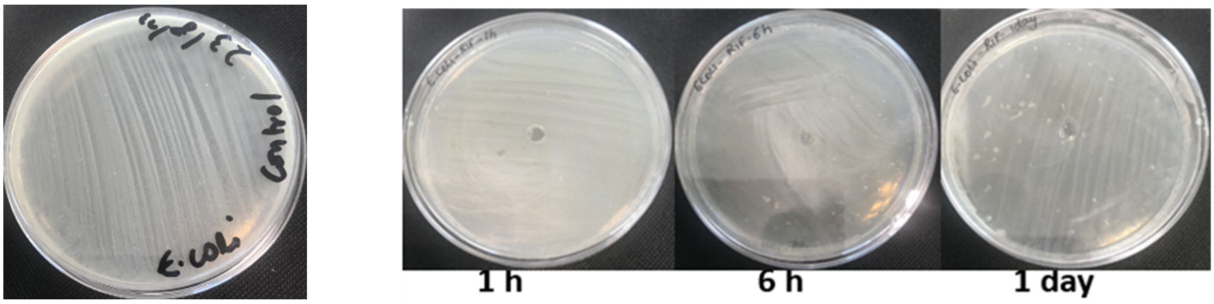

Control PLGA:RIF (8:1)

***E. coli*- Non-direct TSB punch agar**

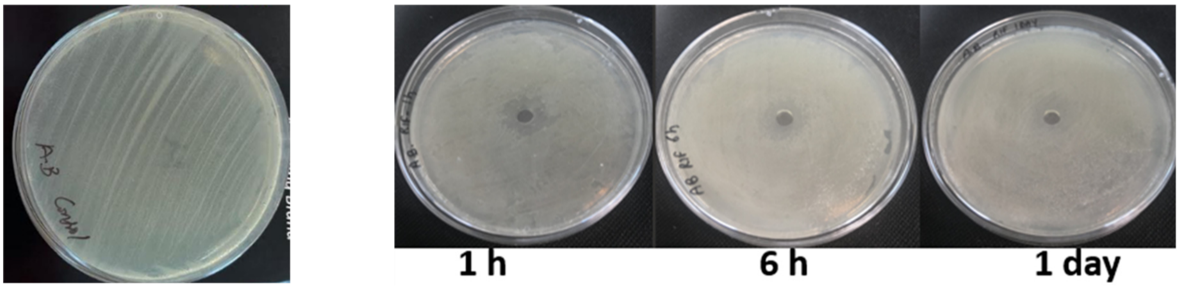

Control PLGA:RIF (8:1)

***A. baumannii*-non-direct TSB punch agar**

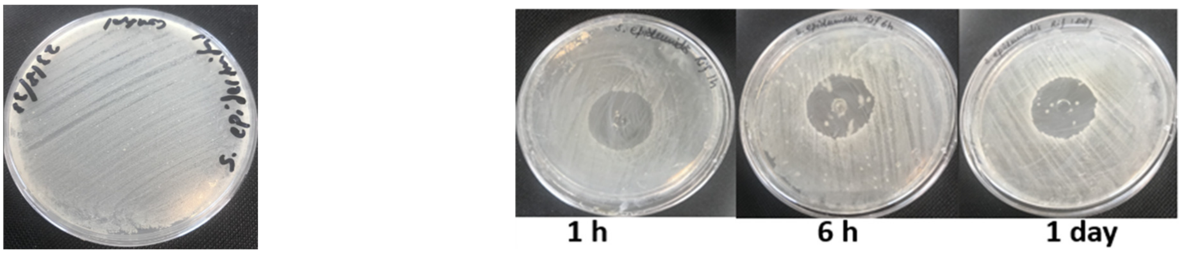

Control **PLGA:RIF (8:1)**

***S. epidermidis*-non-direct TSB punch agar**

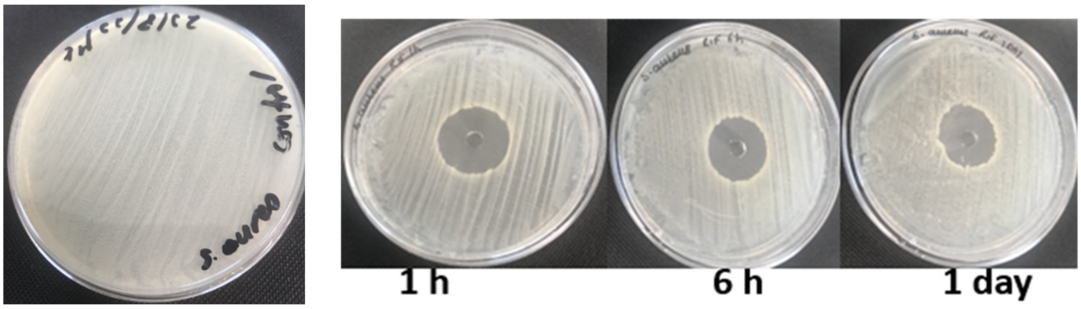

Control **PLGA:RIF (8:1)**

***S. aureus*-non-direct TSB punch agar**

Figure S5: Non-direct antimicrobial activity of the PLGA and PLGA-RIF composites after 1 h, 6 h, and 1 day of immersion in TSB and 50 µm of the supernatant against bacteria.

A

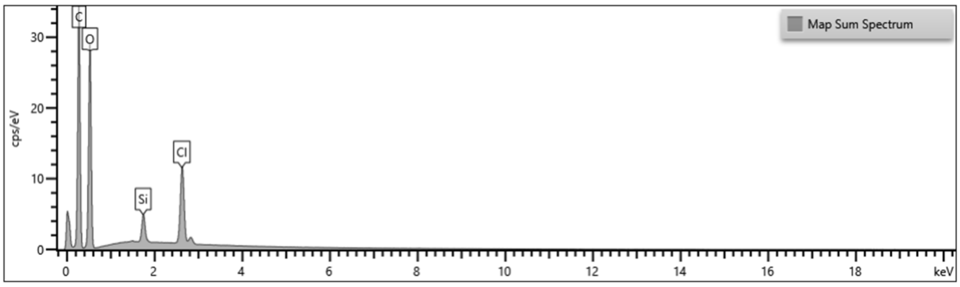

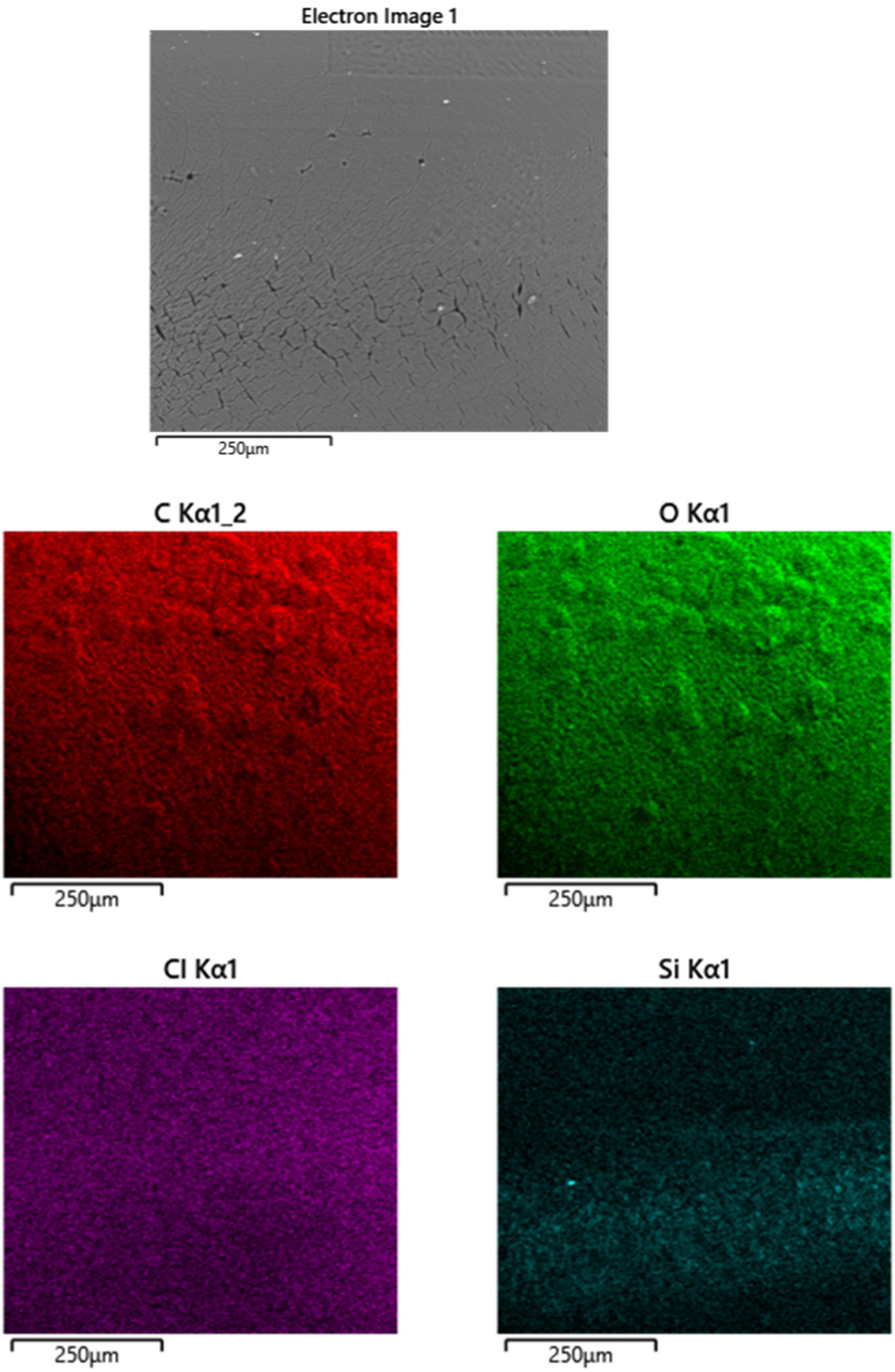
Electron Image 1
250µm
C Kα1_2
250µm
O Kα1
250µm
Cl Kα1
250µm
Si Kα1
250µm

B

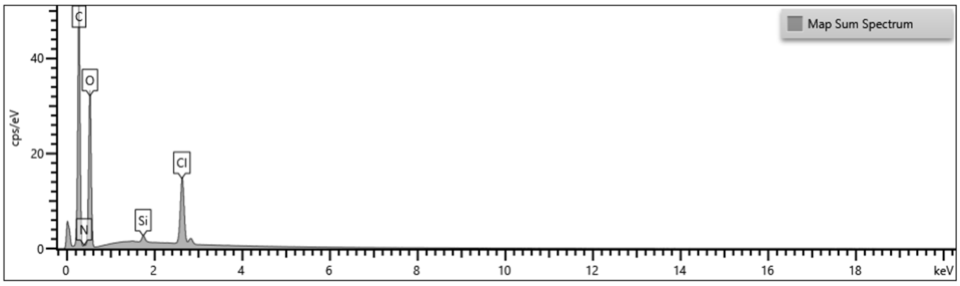

Electron Image 2

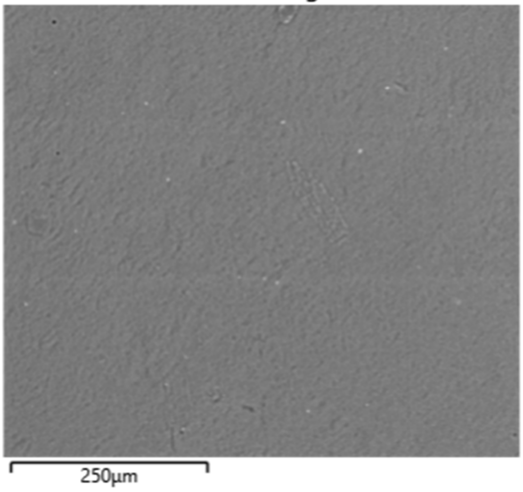

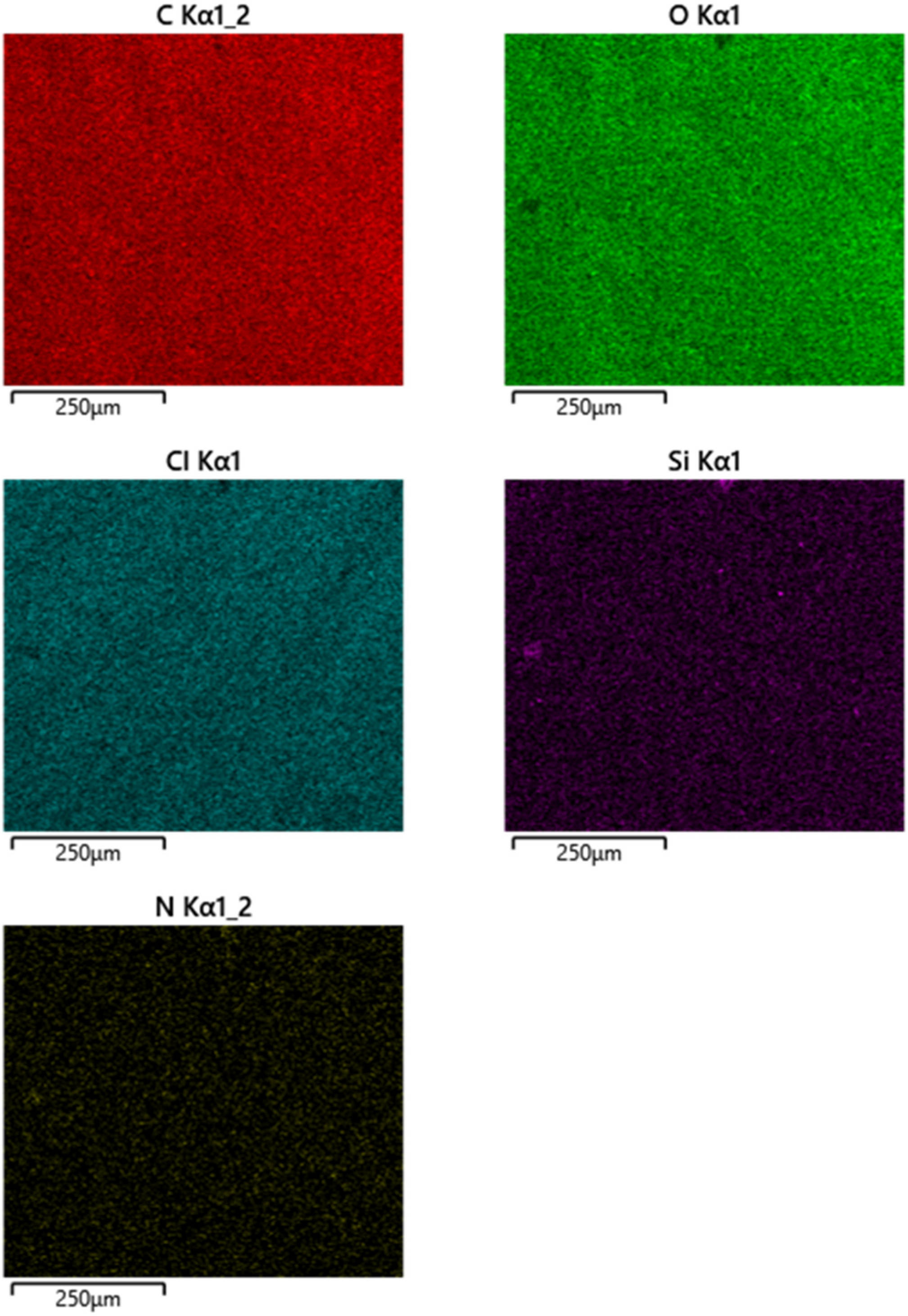
C Kα1_2
250µm
O Kα1
250µm
Cl Kα1
250µm
Si Kα1
250µm
N Kα1_2
250µm

C

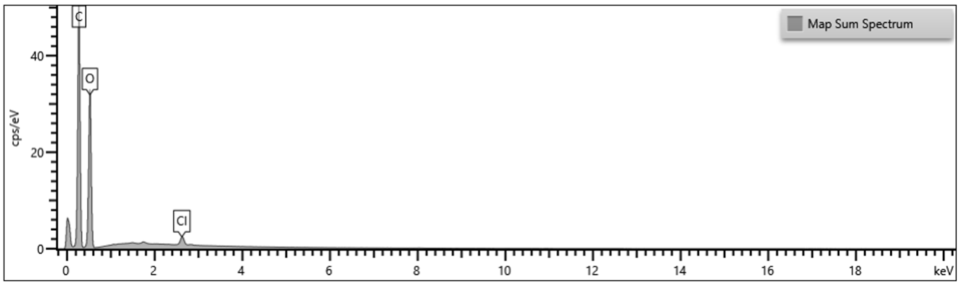
Map Sum Spectrum
cps/eV
C
O
Cl
0
20
40
0
2
4
6
8
10
12
14
16
18
keV

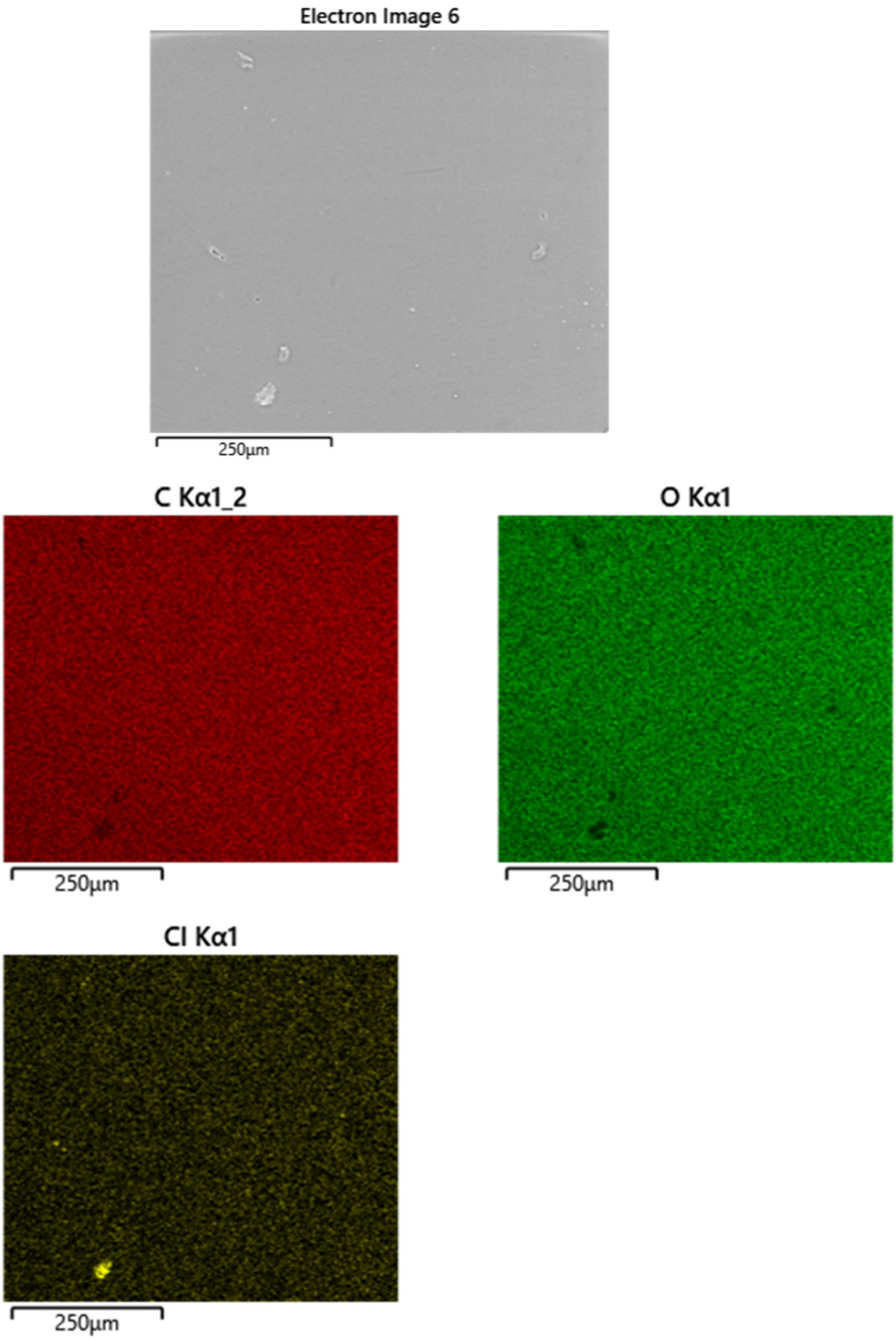
Electron Image 6
250µm
C Kα1_2
250µm
O Kα1
250µm
Cl Kα1
250µm

D

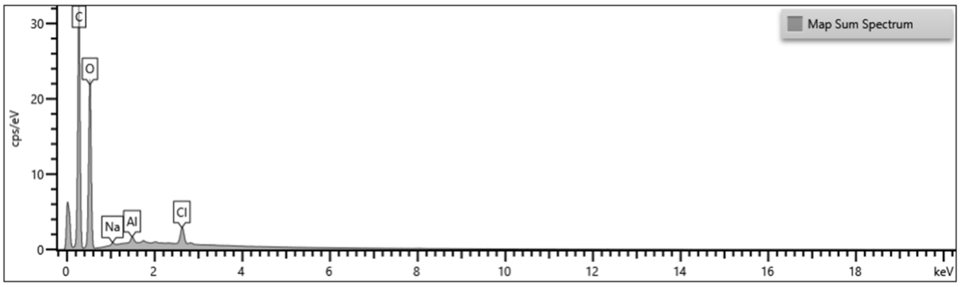
Map Sum Spectrum
cps/eV
30
20
10
0
C
O
Na
Al
Cl
0
2
4
6
8
10
12
14
16
18
keV

Electron Image 5

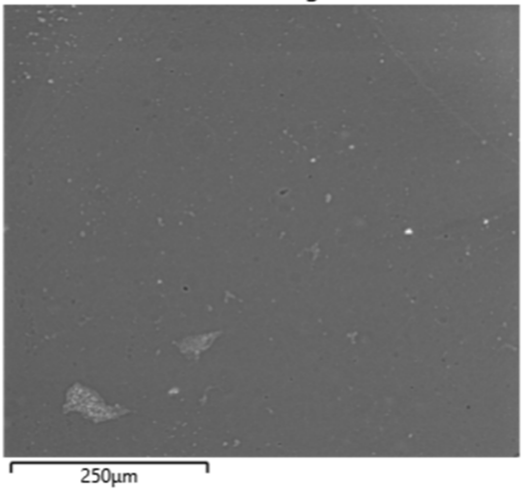

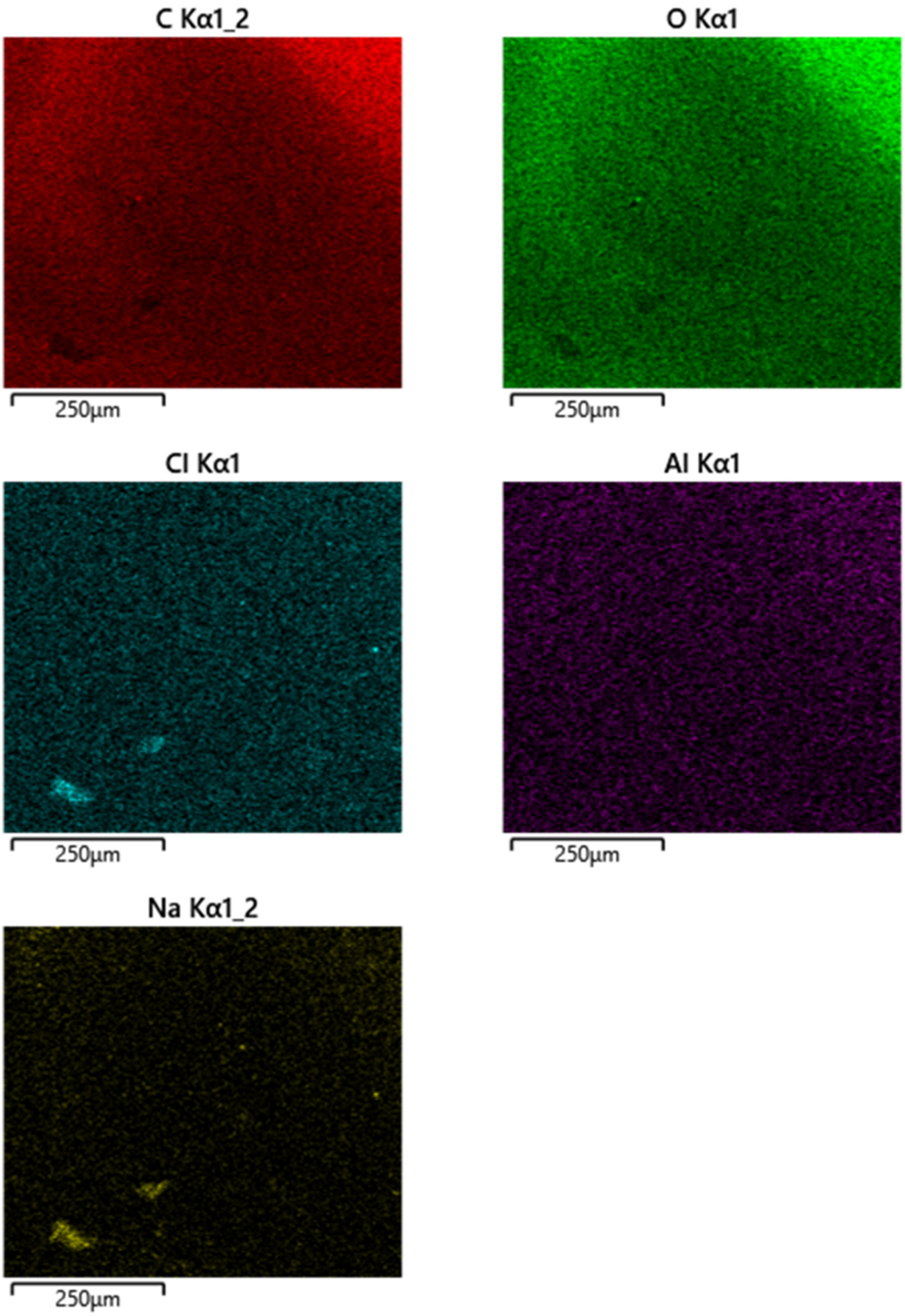

Figure S6: EDS- A) PLGA B) PLGA-RIF C) PLGA Immersed in DMEM D) PLGA-RIF Immersed in DMEM
